# Crown and Bridge-Work

**Published:** 1888-03

**Authors:** F. T. Van Woert

**Affiliations:** Brooklyn, N. Y.


### CROWN AND BRIDGE-WORK.

BY DR. F. T. VAN WOERT, BROOKLYN, N. Y.

READ BEFORE THE CENTRAL DENTAL ASSOCIATION OF NORTHERN NEW JERSEY.

It is with some apprehension that I present the subject of Crown and Bridge-work for your consideration to-night, but if you will kindly bear with me I shall endeavor to convert you to the belief of that old adage, "there is nothing new under the sun," not ignoring the fact that there are those who advance claims to the invention of the great and scientific system known in our profession as Crown and Bridge-work. It seems to me that these so-called inventors are simply elaborators of that which had been discovered many years ago, notwithstanding the fact that the Supreme Court of the United States has endorsed them as being the originators, thereby placing in their hands an exclusive right at the expense of the profession at large. I refer to the International Tooth Crown Co. Now, while Bridge-work is not a part of my practice, I think there are many cases where it could be applied to advantage; but, knowing the unsettled state of these so-called patents, I have kept myself free from the possible chance of a suit at law, and my patients from the annoyance of publicity by having their mouths made a subject for newspaper criticism. To artificial crowns I have given a great deal of time, thought and labor, in the perfecting (not inventing) of the process for their construction.

In searching for something new upon this subject, my attention was called to a work published by Messrs. Carey, Lea & Blanchard, of Philadelphia, in 1835, the title of which is, "A System of Dental Surgery, in three Parts," by Samuel Sheldon Fitch, M. D., Surgeon Dentist, from which I have taken the following extract.

"Of the Manner in which Artificial Teeth Should be Inserted in the Mouth with Ligatures.—These are made of silk, or fine gold or silver wire, and are passed through the tooth or teeth which we fasten in the mouth. When the artificial tooth or teeth are placed in the position we wish them, the ligatures are carried around the two adjoining living teeth, and tied so as to be firm in their places." I simply mention this clause to show that metal was used to fasten artificial teeth by means of surrounding the adjoining living teeth. The fourth method distinctly speaks of fastening several teeth to one or more roots, viz.:

"Of Pivots.—This is the fourth mode of fastening individual artificial teeth, and is, without any doubt, the best mode in which artificial teeth can ever be fastened in the mouth. The pivots are passed into the stumps of teeth, having the crown of a tooth attached to them, and may be done in this way so as to be perfectly firm, and remain useful to the patient for many years. We sometimes fasten several teeth, united in a block, upon one or two stumps. The advantages of this mode over every other is, I believe, now generally admitted. Clasp springs and plates united may be worn with the greatest pleasure, but the stump is the better mode, if sound and firm; if not, pivots should be fastened in them."

This book has also illustrated plates of this class of work, which I had expected to have the pleasure of showing to you this evening, but unfortunately Dr. Parker, to whom they belong, is very ill, and I did not feel like assuming the responsibility of bringing them here without him. Dr. Parker has been approached for the purchase of this book, and as the sum offered was very large, he is suspicious that it was by an agent of the International Tooth Crown Co.

Now that I have given you the most important points of my paper, permit me to present to you my system of crowning teeth. My first care in these operations is the preparation of the parts to be restored. After pursuing the usual course of excavating and cleaning the root or roots, I proceed to fill the apex of each root with a paste made from a formula furnished by Dr. Lord, of New York, viz.:

| | |
|---|---|
| Iodol, | grs. xx. |
| Zinc Oxide, | grs. xxx. |
| Vaseline Car., | Q. S. |

Mix to form a stiff paste.

Over this I place a covering of Dawson's oxy-phosphate, the paste and phosphate occupying about one-third the length of the root. I am speaking now of cases where a dowel or pivot is used, leaving two-thirds for the accommodation of the pin, which is all-sufficient for a crown adjusted in the manner I am about to present. I trim the root down to a proper shape, take a Logan pin and cut from the small end enough of it to allow it to rest upon the phosphate, with the large end protruding about one-sixteenth of an inch. Then from a piece of 22 K., No. 29 or 30 gold plate, I cut to shape, or very near it, a cap through which I drive a punch, resting it upon a block of zinc; this punch being a trifle smaller than the pins, and shaped like them, will allow the pin to pass through the cap about three-quarters of its length; this is to be placed in the root and driven home with a mallet, using for a drift a piece of orange wood, such as is furnished by the dental depots in bundles.

We now have the pin adjusted in the cap to its proper length, and by holding it firmly in place with the drift used, the gold is to be burnished to the form of the root; then removing the pin and cap together, they are soldered with 20 K. solder over a Bunsen flame or spirit lamp. After soldering, replace the pin and cap upon the root, and with a plug finishing bur (sugar loaf, No. 243) trim the cap to the exact size of the root, and cut the pin from the upper surface so that it is flush with the top of the cap. Now select a plate tooth of the proper shape, size, and color, back it with 22 K. gold and grind it to fit the cap. Dry the parts and wax the tooth fast with ordinary base wax, remove and invest in pumice and plaster, about equal parts, trimming it down so that there is a covering of about one-fourth of an inch all round. The wax can then be removed with the point of a knife. Place it over or in the fire until the piece is thoroughly dried, then flow upon it 20 K. solder to the thickness required. After removing from the investment, the gold work should be trimmed and contoured with suitable corundum wheels, and polished with Dr. E. Parmly Brown's moose-hide points. I then barb the pin with a common knife, cut a few irregular grooves in the root, and set the crown with Dawson's Cement. This last, the operation of setting, is very pleasing to the operator, as the crown will go to its place without the least difficulty; in fact, it is almost impossible to get it wrong after once properly adjusting or fitting it.

The advantages I claim in this over other systems are the following: First—The very little skill required to make a true union between the crown and root. Second—The simplicity of the whole operation, and the comparatively short time required to complete a crown of this kind—about one hour from the time your patient takes the chair until he is ready to leave it; there is no pain connected with the operation, and very little, if any, fatigue. Third—You have in this crown all the virtues of the band-cap crown, besides its better appearance, which your patient will concede at once, and you can dismiss him feeling perfectly sure of the stability of your work, and not apprehensive of a visit from him in a short time, with the declaration, perhaps made before waiting patients, that the tooth is off.

Having thus briefly spoken of my method of crowning the six front teeth with the combination of gold and porcelain, permit me for a few moments to call your attention to the crowning of bicuspids and molars. We all know that these cases vary considerably; where, in my judgment, it is practical, I use in preference the gold band with a porcelain tip, but in many cases I find it impossible to get the required strength in this manner, and as a consequence resort to the all-gold crown, which we know is ever reliable and can be depended upon, if properly adjusted, and in the construction of this I claim a little originality.

After preparing the root I make of 22 K. gold, No. 28 plate, a band of sufficient width to allow a perfect clearance of the crown floor. I select a natural tooth (of which I have a sufficient number at my command) that will give me the required contour. I then take the thinnest platinum I can get, which is about like the rolled gold that is sometimes used in finishing fillings, and burnish this into and around the crown of the natural tooth, trimming it down with a pair of curved scissors. I throw into it a little powdered borax and scrap gold of 22 K., and hold it over a Bunsen flame until it has melted and flowed, adding to it a sufficient quantity to completely fill this platinum mould. Then with a No. 4 Grobett file I cut the lower surface perfectly straight. The ring having been filed in like manner, I place it upon the crown and solder with 18 K. solder. I then take a piece of pine wood, and with the gold mallet drive the band into the wood to the floor of the crown; then with my penknife I trim off the outside of the wood so that I can get at the crown, and with a file of a suitable cut I file off the platinum and surplus gold, using plug finishing burs in the crevices and curves that the files will not touch. This leaves me a crown of all 22 K. gold, and solid from the root to its top, which I set in the usual manner.

